# Patterns of stressful life events and polygenic scores for five mental disorders and neuroticism among adults with depression

**DOI:** 10.1038/s41380-024-02492-x

**Published:** 2024-04-04

**Authors:** Jacob J. Crouse, Shin Ho Park, Enda M. Byrne, Brittany L. Mitchell, Jan Scott, Sarah E. Medland, Tian Lin, Naomi R. Wray, Nicholas G. Martin, Ian B. Hickie

**Affiliations:** 1 Brain and Mind Centre, Faculty of Medicine and Health, University of Sydney, Sydney, NSW Australia; 2 Institute for Molecular Bioscience, The University of Queensland, Brisbane, QLD Australia; 3 Child Health Research Centre, The University of Queensland, Brisbane, QLD Australia; 4 Mental Health and Neuroscience Program, QIMR Berghofer Medical Research Institute, Brisbane, QLD Australia; 5 Academic Psychiatry, Institute of Neuroscience, Newcastle University, Newcastle, UK; 6 Norwegian University of Science and Technology, Trondheim, Norway; 7 Université de Paris, Paris, France; 8 Queensland Brain Institute, The University of Queensland, Brisbane, QLD Australia; 9 Department of Psychiatry, University of Oxford, Oxford, UK; 10 Oxford Big Data Institute, Li Ka Shing Centre for Health Information and Discovery, University of Oxford, Oxford, UK

**Keywords:** Genetics, Depression

## Abstract

The dominant (‘general’) version of the diathesis-stress theory of depression views stressors and genetic vulnerability as independent risks. In the Australian Genetics of Depression Study (*N* = 14,146; 75% female), we tested whether polygenic scores (PGS) for major depression, bipolar disorder, schizophrenia, anxiety, ADHD, and neuroticism were associated with reported exposure to 32 childhood, past-year, lifetime, and accumulated stressful life events (SLEs). In false discovery rate-corrected models, the clearest PGS-SLE relationships were for the ADHD- and depression-PGSs, and to a lesser extent, the anxiety- and schizophrenia-PGSs. We describe the associations for childhood and accumulated SLEs, and the 2–3 strongest past-year/lifetime SLE associations. Higher ADHD-PGS was associated with all childhood SLEs (emotional abuse, emotional neglect, physical neglect; ORs = 1.09–1.14; p’s < 1.3 × 10^−^^5^), more accumulated SLEs, and reported exposure to sudden violent death (OR = 1.23; *p* = 3.6 × 10^−^^5^), legal troubles (OR = 1.15; *p* = 0.003), and sudden accidental death (OR = 1.14; *p* = 0.006). Higher depression-PGS was associated with all childhood SLEs (ORs = 1.07–1.12; p’s < 0.013), more accumulated SLEs, and severe human suffering (OR = 1.17; *p* = 0.003), assault with a weapon (OR = 1.12; *p* = 0.003), and living in unpleasant surroundings (OR = 1.11; *p* = 0.001). Higher anxiety-PGS was associated with childhood emotional abuse (OR = 1.08; *p* = 1.6 × 10^−^^4^), more accumulated SLEs, and serious accident (OR = 1.23; *p* = 0.004), physical assault (OR = 1.08; *p* = 2.2 × 10^−^^4^), and transportation accident (OR = 1.07; *p* = 0.001). Higher schizophrenia-PGS was associated with all childhood SLEs (ORs = 1.12–1.19; p’s < 9.3^−^^8^), more accumulated SLEs, and severe human suffering (OR = 1.16; *p* = 0.003). Higher neuroticism-PGS was associated with living in unpleasant surroundings (OR = 1.09; *p* = 0.007) and major financial troubles (OR = 1.06; *p* = 0.014). A reversed pattern was seen for the bipolar-PGS, with lower odds of reported physical assault (OR = 0.95; *p* = 0.014), major financial troubles (OR = 0.93; *p* = 0.004), and living in unpleasant surroundings (OR = 0.92; *p* = 0.007). Genetic risk for several mental disorders influences reported exposure to SLEs among adults with moderately severe, recurrent depression. Our findings emphasise that stressors and diatheses are inter-dependent and challenge diagnosis and subtyping (e.g., reactive/endogenous) based on life events.

## Introduction

The diathesis-stress model is the dominant aetiological theory of depressive disorders [1]. Multiple versions of this model have been proposed, which each describe different forms of relationship between stressors and diatheses and have distinct implications for understanding, preventing, and treating depression [2]. While several studies support a complex, inter-dependent relationship between stressors and diatheses [3, 4], the ‘general’ version of the model (which predicts that people with a higher genetic vulnerability for depression should have a lower load of stressors compared to people with lower genetic vulnerability) remains the most popular theory of the aetiology of depression [5]. Specific typologies of depression have been proposed based on this idea [6]. For example, people with so-called ‘endogenous’ depression have been theorised to have high genetic or biologic load but low environmental load, while cases with ‘reactive’ depression are theorised to have low genetic or biologic load but high environmental load.

The general version of the diathesis-stress model of depression and the proposed distinction between ‘endogenous’ and ‘reactive‘ subtypes fail to adequately consider that stressors and genetic diatheses are often inter-related. Indeed, there is some evidence that exposure to SLEs may be partly heritable [7], with twin studies showing that a class of SLEs for which individuals might play a contributory role seem to be influenced by genetic factors, albeit with modest heritability estimates [8–11]. This class of SLEs are referred to as ‘dependent’ or ‘personal’ SLEs (e.g., being laid off from one’s job), while SLEs that are more likely to be a function of random chance (e.g., exposure to a natural disaster) are referred to as ‘independent’ or ‘nonpersonal’ SLEs. Three models are commonly used to explain such ‘gene-environment correlations’. The ‘active’ model views people with genetic vulnerability to depression as being more likely to generate stress by selecting themselves into environments that have higher risk of exposure to stressors, such as a dysfunctional intimate relationship [12–15]. By contrast, the ‘passive’ model suggests that since mental disorders are heritable, genetic vulnerability may be a (noncausal) marker of a parent’s psychopathology, and a parent’s mental health problems may be the proximal cause of increased risk of exposure to SLEs in offspring. Third, the ‘evocative’ model proposes that genetically-influenced traits (e.g., stubbornness) evoke predictable patterns of response from the social environment (e.g., berated by a family member). Given these theoretical distinctions and the plausible contribution of individual factors (e.g., genes) to reported exposure to SLEs, a coherent aetiologic theory of depression should consider the influence genes have on exposure (or reporting of exposure) to SLEs [16].

Recent genetic studies have tested whether people with a higher polygenic score (PGS) for depression are more likely to develop depression after exposure to SLEs, with conflicting findings [3, 4, 17–19]. Very few studies have examined whether PGSs for depression or other mental disorders (and correlated traits) are associated with reported exposure to SLEs. A study of cases with depression in *iPSYCH2012* reported a small relationship between higher depression-PGS and increased risk of exposure to at least one SLE after age 10^18^. A case-control study reported a small association between higher depression-PGS and the number of SLEs individuals were exposed to, but only in cases with depression [20]. Population-based studies have also investigated this question. A study from *Generation Scotland* reported a small association between a higher depression-PGS and the number of reported SLEs [21]. By contrast, a study of older adults in the *Health and Retirement Study* reported that the depression-PGS was unrelated to exposure to SLEs [22]. A handful of studies have examined whether multiple PGSs for mental disorders are mutually associated with reporting of SLEs. A study of a population-based cohort of women found that higher PGSs for major depression and ADHD were associated with higher likelihood of reporting exposure to childhood abuse, while PGSs for neuroticism, schizophrenia, bipolar disorder, and autism spectrum disorder (ASD) were associated with physical and emotional (but not sexual) abuse [23]. A study of the *Twins Early Development Study* reported a higher likelihood of retrospective childhood trauma in those with higher PGSs for ASD and PTSD, but not major depression, ADHD, bipolar disorder, schizophrenia, neuroticism, or anxiety [24].

No study of people with depression has investigated the mutual impact of PGSs for depression and other mental disorders on reported exposure to SLEs. Accordingly, we use a cohort study of adults with depression to examine associations between reported exposure to SLEs and PGSs for five major mental disorders (depression, bipolar disorder, schizophrenia, anxiety disorder, ADHD) and a related trait (neuroticism). We focus on these traits for four reasons. First, they are relevant to diathesis-stress models of depression and have plausible links to the experience, reporting, and/or exposure to SLEs (particularly PGS for neuroticism, anxiety, and depression). Second, there is face validity in the idea that behavioural manifestations of genetic liability to these six traits may increase the likelihood of being exposed to SLEs (particularly PGS for schizophrenia and ADHD). Third, these PGS are relatively well-powered, with SNPs identified by suitably-powered GWAS. Fourth these six traits are central to our ‘tripartite’ model about differential pathways to depressive disorders [25, 26]. Building on the literature, we hypothesise that higher depression-PGS will be associated with higher likelihood of reported exposure to SLEs, while investigation of the other PGS-SLE associations are exploratory.

## Materials and methods

### Participants and study design

The *Australian Genetics of Depression Study* (AGDS) is a volunteer cohort study of adults with depression. Recruitment procedures and cohort characteristics have been detailed elsewhere [27]. Participants joined AGDS after replying to a media campaign or a letter from the Australian Government’s Department of Human Services, sent to Australian residents who had received ≥ 4 prescriptions of any of the 10 commonest antidepressant medications in Australia in the past 4.5 years. 100,000 letters were sent across two waves (2016, 2017) and more participants were recruited via media appeal (85.7%) than prescription history invitation (14.3%). Participants completed an online survey about mental/physical health, treatment, and social, behavioural, and environmental factors [27]. The sample are highly educated (32% with a degree, 24% with a postgraduate degree) and > 75% have contributed a saliva sample using a mail-out ‘spit-kit’ from which DNA was extracted and processed.

The study was approved by the QIMR Berghofer Medical Research Institute Human Research Ethics Committee in Brisbane, Australia. Written informed consent was obtained from all participants. This report followed the Strengthening the Reporting of Observational Studies in Epidemiology (STROBE) guidelines [28].

### Stressful life events (SLEs)

#### Childhood SLEs

Participants indicated whether they experienced any of three types of SLEs in childhood: (1) emotional abuse (e.g., often being told you were no good; yelled at in a scary way; threatened, ignored, or stopped from making friends); (2) emotional neglect (e.g., often not being shown affection; not being given encouragement or support); or (3) physical neglect (e.g., often not being given enough to eat or drink, appropriate clothing, shelter, medical care, education, supervision, or a safe home environment). Three possible responses (no, yes, unsure) were recoded (1 = yes; 0 = no/unsure).

#### Lifetime SLEs

Estimated using the Life Events Checklist for DSM-5 [29]. Participants indicated whether they had experienced any of the following SLEs: (1) natural disaster (e.g., flood, cyclone, tornado, earthquake); (2) fire or explosion; (3) transportation accident (e.g., car accident, boat accident, train wreck, plane crash); (4) serious accident at work, home, or during recreational activity; (5) exposure to toxic substances (e.g., dangerous chemicals, radiation); (6) physical assault (e.g., being attacked, hit, slapped, kicked, beaten up); (7) assault with a weapon (e.g., being shot, stabbed, threatened with a knife, gun, bomb); (8) sexual assault (e.g., rape, attempted rape, made to perform any type of sexual act through force or threat of harm); (9) other unwanted/uncomfortable sexual experience; (10) combat or exposure to a war-zone (in the military or as a civilian); (11) captivity (e.g., being kidnapped, abducted, held hostage, prisoner of war); (12) life-threatening illness or injury; (13) severe human suffering; (14) sudden violent death (e.g., homicide, suicide); (15) sudden accidental death; (16) serious injury, harm or death you caused to someone else; or (17) any other very stressful event or experience. Six possible responses (happened to me, witnessed it, learned about it, part of my job, not sure, doesn’t apply) were recoded (1 = happened to me; 0 = all other responses).

#### Past-year SLEs

Estimated using 12 items adapted from the List of Threatening Experiences [30]. Participants indicated whether they experienced any of the following in the past 12 months: (1) divorce; (2) marital separation; (3) broken engagement/steady relationship; (4) separation from other loved one or close friend; (5) serious illness or injury; (6) serious accident (not involving personal injury); (7) burgled or robbed; (8) laid off or sacked from job; (9) other serious difficulties at work; (10) major financial problems; (11) legal troubles or involvement with police; or (12) living in unpleasant surroundings. Two responses were possible (0 = no; 1 = yes).

#### Cumulative SLEs

We summed the number of SLEs across four categories: childhood (range = 0–3); lifetime (0–17); past-year (0–12); and overall (i.e., summing across the other three categories; 0–32). Four SLEs had conceptual overlap across the checklists: ‘serious accident’ (lifetime) with ‘serious accident’ (past-year) and ‘life-threatening illness or injury’ (lifetime) with ‘serious illness or injury’ (past-year). To avoid double-counting, we discarded the past-year version when creating the cumulative SLE variable.

### Polygenic scores (PGS)

Genotyping was done using the Illumina Global Screening Array V2. Samples were merged with the 1000 Genomes project samples [31] and genetic principal components (PCs) were calculated using a set of single-nucleotide polymorphisms (SNPs) not in linkage disequilibrium. Pre-imputation quality control was done using PLINK 1.9 [32, 33], including removing SNPs with a minor allele frequency < 0.005, SNP call rate < 97.5%, and identification of participants with genetic similarity [34] to a European reference group (> 4 SD from Ancestry Principal Components [PCs] PC1/PC2 centroid) and Hardy-Weinberg equilibrium (*p* < 1 × 10^−^^6^), before imputation using the Haplotype Reference Consortium 1.1 reference panel [35]. Over 95% of AGDS is of European ancestry and PGS were created for those with European ancestry only. PCA plots of ancestry principal components are shown in Supplementary Fig. 1. Other than requiring data for PGS and SLEs, no inclusion/exclusion criteria was applied for this study.

Summary statistics from recent genome-wide association studies (GWAS) were used to identify SNPs associated with major depression [36], bipolar disorder [37], schizophrenia [38], anxiety [39], ADHD [40], and neuroticism [41]. *SBayesR* [42], a Bayesian PGS method, was used to generate allele weights for each PGS. The posterior SNP effects for each disorder/trait were used to generate PGS for each participant using the *PLINK* score function [32]. As SNPs from AGDS participants have been used in depression GWAS, our depression-PGS was calculated with AGDS participants excluded. Each PGS was standardised across the entire QIMR genetic datasets (*N* > 60,000); the mean and standard deviations (SD) of each PGS are: major depression (*M* = 0.29; SD = 0.98); bipolar disorder (*M* = 0.11; SD = 1.00); schizophrenia (*M* = 0.07; SD = 1.00); anxiety disorder (*M* = 0.12; SD = 0.99); ADHD (*M* = 0.07; SD = 1.00); and neuroticism (*M* = 0.10; SD = 0.96). PGS were standardised for analysis. Effect sizes are interpretable as reflecting a one SD increase/decrease.

### Statistical analysis

Analyses were conducted using R (version 4.2.2) [43]. Pearson’s product moment correlation was used to examine univariate relationships between each PGS (continuous) and the 32 SLEs (dichotomous). Logistic regression was used to model associations between SLEs and PGSs, while linear regression was used to model associations between the number of SLEs in each epoch (childhood, lifetime, past-year) and accumulated (range 0–32) and the PGS; models were adjusted for age and sex. The data were inspected before running analyses to confirm they met the assumptions of the statistical tests. The sample size was not pre-determined for this specific study but reflects participants with data on PGS and SLEs. To control for multiple comparisons in the multivariate analyses, we report uncorrected *p*-values and false discovery rate (FDR) significant *p*-values, calculated using the Benjamini-Hochberg (BH) procedure [44]: (1) sort *p*-values from multivariable models in ascending order; (2) assign a rank to each *p*-value; (3) calculate each *p*-value’s BH critical value using the formula (i/m)Q, where: *i* = rank of *p*-value, *m* = number of tests, and *Q* = false discovery rate (5%); and (4) find largest *p*-value that is less than the critical value and designate smaller *p*-values as “significant”. Model outputs are reported in Supplementary Tables 5–40.

## Results

### Demographic and clinical data

From an available cohort of 20,680 individuals (75% female; mean [SD] age 42.8 years [15.3 years]), data passing quality control were available for 14,146 participants and included as our analytic sample. 75% were female and the mean [SD] age was 44.0 years [15.3] (range 18–90). Using DSM-5 criteria, 88% met criteria for a lifetime major depressive episode. Descriptive information about the sample is provided in Table 1.

**Table 1:** Socio-demographic characteristics of the sample.

Total N	14,146
**Age, years**	
Mean (SD)	43.95 (15.27)
Range	18–90
**Sex** ^**a**^	
Female	10,561 (75%)
Male	3572 (25%)
Unspecified	13 ( < 1%)
**Marital status**	
Married or de facto relationship	7682 (54%)
Separated or divorced	2159 (15%)
Widowed	249 (2%)
Never married	4025 (29%)
Information not provided	31 ( < 1%)
**Education**	
Postgraduate	3927 (28%)
Degree	4867 (35%)
Certificate or diploma	3379 (24%)
Senior high school	1110 (8%)
Junior high school or less	830 (6%)
No formal education	6 ( < 1%)
Information not provided	27 ( < 1%)

### Reported exposure to SLEs

Numbers of participants reporting exposure to each of the 32 SLEs are presented in Supplementary Table 1. The three commonest were emotional abuse (53%) and emotional neglect (46%) in childhood and lifetime other unwanted or uncomfortable sexual experience (34%), while the least common was combat or exposure to a warzone (< 1%).

### Childhood SLEs and PGSs

**Fig. 1:**
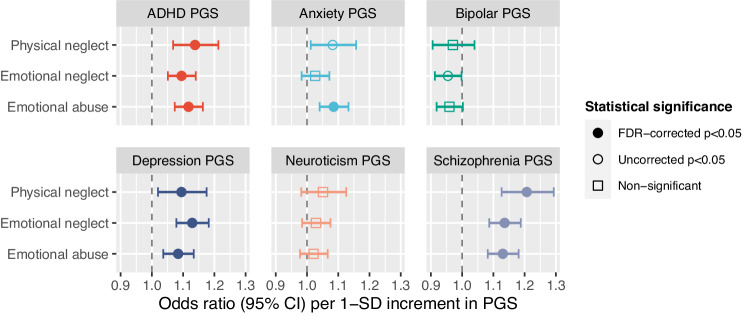
Childhood SLEs and PGS for attention-deficit/hyperactivity disorder, anxiety, bipolar disorder, depression, neuroticism, and schizophrenia. Coefficients are presented grouped by PGS (not SLE). The *y*-axis variables are the outcome (y variables) for three regression models in which the six PGS were fitted alongside age and sex (x variables).

As shown in Fig. 1, higher PGSs for ADHD, schizophrenia, and depression were associated with a higher likelihood of reported exposure to all childhood SLEs (FDR-corrected p’s < 0.05). Higher anxiety-PGS was associated with higher odds of reported exposure to emotional abuse in childhood (FDR-corrected *p* < 0.05). The bipolar- and neuroticism-PGS were not associated with reported exposure to any childhood-SLE.

### Lifetime SLEs and PGSs

**Fig. 2:**
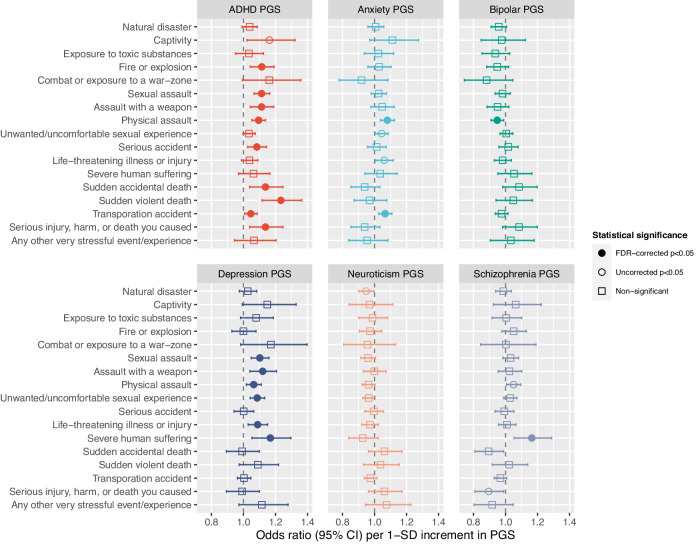
Lifetime SLEs and PGSs for attention-deficit/hyperactivity disorder, anxiety, bipolar disorder, depression, neuroticism, and schizophrenia. Coefficients are presented grouped by PGS (not SLE). The *y*-axis variables are the outcome (*y* variables) for 17 regression models in which the six PGS were fitted alongside age and sex (x variables).

As shown in Fig. 2, under FDR-correction there were no significant relationships between any lifetime-SLE and the neuroticism-PGS. The bipolar-PGS was associated a lower likelihood of reported exposure to physical assault (OR = 0.95 [95% CI,0.91–0.99]; *p* = 0.014; FDR-corrected *p* < 0.05). Higher schizophrenia-PGS was related to a higher odds of severe human suffering (OR = 1.16 [1.05–1.29]; *p* = 0.003; FDR-corrected *p* < 0.05). Higher anxiety-PGS was associated with higher likelihood of physical assault (OR = 1.08 [1.04–1.13]; *p* < 0.001; FDR-corrected *p* < 0.05) and transportation accident (OR = 1.07 [1.03–1.11]; *p* = 0.001; FDR-corrected *p* < 0.05). Higher depression-PGS was associated with higher odds of physical assault (OR = 1.06 [1.02–1.11]; *p* = 0.006; FDR-corrected *p* < 0.05), unwanted/uncomfortable sexual experience (OR = 1.09 [1.04–1.13]; *p* < 0.001; FDR-corrected *p* < 0.05), sexual assault (OR = 1.10 [1.05–1.16]; *p* < 0.001; FDR-corrected *p* < 0.05), severe human suffering (OR = 1.17 [1.05–1.30]; *p* = 0.003; FDR-corrected *p* < 0.05), life-threatening illness or injury (OR = 1.09 [1.03–1.15]; *p* = 0.003; FDR-corrected *p* < 0.05), and assault with a weapon (OR = 1.12 [1.04–1.21]; *p* = 0.003; FDR-corrected *p* < 0.05). Finally, higher ADHD-PGS was associated with higher likelihood of sudden violent death (OR = 1.23 [1.12–1.36]; *p* < 0.001; FDR-corrected *p* < 0.05), sudden accidental death (OR = 1.14 [1.04–1.25]; *p* = 0.006; FDR-corrected *p* < 0.05), sexual assault (OR = 1.11 [1.07–1.16]; *p* < 0.001; FDR-corrected *p* < 0.05), serious accident (OR = 1.08 [1.03–1.14]; *p* = 0.004; FDR-corrected *p* < 0.05), physical assault (OR = 1.09 [1.05–1.14]; *p* < 0.001; FDR-corrected *p* < 0.05), fire or explosion (OR = 1.11 [1.04–1.19]; *p* = 0.001; FDR-corrected *p* < 0.05), assault with a weapon (OR = 1.11 [1.04–1.19]; *p* = 0.001; FDR-corrected *p* < 0.05), and transportation accident (OR = 1.05 [1.01–1.09]; *p* = 0.018; FDR-corrected *p* < 0.05).

### Past-year SLEs and PGSs

**Fig. 3:**
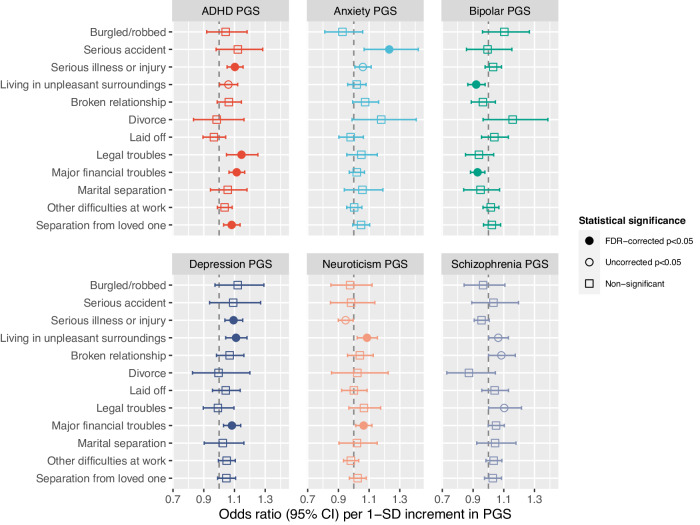
Past-year SLEs and PGSs for ADHD, anxiety, bipolar disorder, depression, neuroticism, and schizophrenia. Coefficients are presented grouped by PGS (not SLE). The *y*-axis variables are the outcome (*y* variables) for 12 regression models in which the six PGS were fitted alongside age and sex (*x* variables).

As presented in Fig. 3, under FDR-correction the schizophrenia-PGS was not significantly associated with any past-year SLE. Higher anxiety-PGS was associated with a higher likelihood of serious accident (OR = 1.23 [95% CI,1.07–1.42]; *p* = 0.004; FDR-corrected *p* < 0.05). Higher neuroticism-PGS was associated with higher likelihood of living in unpleasant surroundings (OR = 1.09 [1.02–1.15]; *p* = 0.007; FDR-corrected *p* < 0.05) and major financial troubles (OR = 1.06 [1.01–1.12]; *p* = 0.014; FDR-corrected *p* < 0.05). Higher depression-PGS was associated with higher odds of serious illness or injury (OR = 1.09 [1.04–1.15]; *p* < 0.001; FDR-corrected *p* < 0.05), major financial troubles (OR = 1.08 [1.03–1.14]; *p* = 0.002; FDR-corrected *p* < 0.05), and living in unpleasant surroundings (OR = 1.11 [1.04–1.18]; *p* = 0.001; FDR-corrected *p* < 0.05). By contrast, higher bipolar-PGS was associated lower likelihood of major financial troubles (OR = 0.93 [0.88–0.98]; *p* = 0.004; FDR-corrected *p* < 0.05) and living in unpleasant surroundings (OR = 0.92 [0.87–0.98]; *p* = 0.008; FDR-corrected *p* < 0.05). Finally, higher ADHD-PGS was associated with higher odds of serious illness or injury (OR = 1.10 [1.05–1.16]; *p* < 0.001; FDR-corrected *p* < 0.05), separation from loved one (OR = 1.08 [1.03–1.14]; *p* = 0.002; FDR-corrected *p* < 0.05), major financial troubles (OR = 1.11 [1.06–1.17]; *p* < 0.001; FDR-corrected *p* < 0.05), and legal troubles (OR = 1.15 [1.05–1.25]; *p* = 0.003; FDR-corrected *p* < 0.05).

### Cumulative SLEs and PGSs

**Fig. 4:**
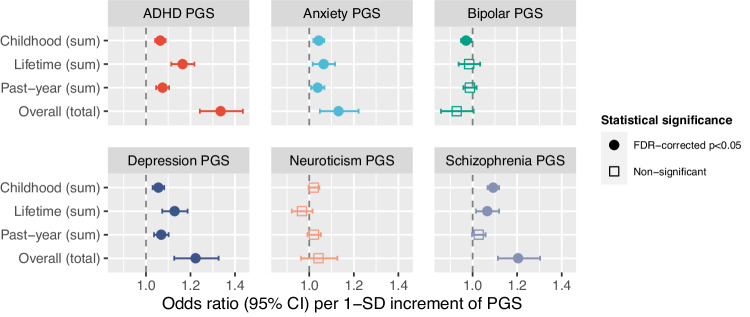
Cumulative SLEs and PGSs for ADHD, anxiety, bipolar disorder, depression, neuroticism, and schizophrenia. For simplicity, coefficients are presented grouped by PGS (not SLE). The *y*-axis variables are the outcome (*y* variables) for four separate regression models in which the six PGS were fitted alongside age and sex (x variables).

As shown in Fig. 4, the neuroticism-PGS was not significantly related to any cumulative SLE variable. The schizophrenia-PGS was related to more SLEs in childhood, the lifetime, and overall (ORs = 1.09–1.20; FDR-corrected p’s < 0.05). Higher ADHD-PGS was related to more childhood, lifetime, past-year, and cumulative SLEs (ORs = 1.07–1.36; FDR-corrected p’s < 0.05), as was the depression-PGS (ORs = 1.05–1.24; FDR-corrected p’s < 0.05) and anxiety-PGS (OR = 1.04–1.13; FDR-corrected p’s < 0.05). By contrast, higher bipolar-PGS was related to fewer reported SLEs in childhood (OR = 0.97 [0.95–0.99]; *p* = 0.01; FDR-corrected *p* < 0.05).

### Sensitivity analyses

#### Depressive episodes

Higher depression-PGS is associated with more depressive episodes; people experiencing more episodes may be more likely to be subsequently exposed to SLEs; and more episodes may lead to hopelessness that biases reporting. The depression-, schizophrenia-, anxiety-, neuroticism-, and ADHD-PGS were each associated with more depressive episodes and more SLEs, and the number of depressive episodes was associated with all childhood and most past-year and lifetime SLEs (Supplementary Tables 2–4). When including number of depressive episodes as a covariate, the PGS-SLE associations were slightly attenuated but qualitatively similar (Supplementary Tables 5–40).

#### Non-psychiatric PGS

It is possible that the genetic relationships with SLEs are not specific to psychiatric PGS. In a post-hoc analysis, we chose six control PGS, three with minimal genetic correlations (rG = 0.02 0.04) with depression (hip circumference, rheumatoid arthritis, type 2 diabetes [T2D]) and three with stronger genetic correlations (rG = 0.15–0.16) with depression (lung cancer, asthma, coronary artery disease [CAD]). These non-psychiatric PGS were standardised against an independent control sample (ASPREE). We ran the same regression models but with the psychiatric PGS removed and the ‘control’ PGS fitted together (Supplementary Figs. 2–5).

Starting with the traits more strongly genetically correlated with depression, the lung cancer-PGS was associated with three SLEs: ‘serious illness or injury’ (OR = 1.06; *p* = 0.012); major financial problems (OR = 1.06; *p* = 0.008); and sudden violent death (OR = 1.11; *p* = 0.036). The asthma-PGS was associated with eight SLEs, with the five strongest associations for: count of accumulated SLEs (OR = 1.12; *p* = 0.002); major financial problems (OR = 1.08; *p* = 0.011); living in unpleasant surroundings (OR = 1.07; *p* = 0.016); unwanted sexual experience (OR = 1.06; *p* = 0.004); and physical assault (OR = 1.06; *p* = 0.007). The CAD-PGS was associated with six SLEs, with the five strongest associations for: serious injury, harm, or death you caused to someone else (OR = 1.17; *p* = 0.014); legal troubles or involvement with police (OR = 1.14; *p* = 0.003); count of accumulated SLEs (OR = 1.09; *p* = 0.016); count of lifetime SLEs (1.06; *p* = 0.016); and serious illness or injury (OR = 1.06; *p* = 0.020).

Regarding the traits minimally correlated with depression, the hip circumference-PGS was associated with three reported SLEs: fire or explosion (OR = 0.93; *p* = 0.037); unwanted sexual experience (OR = 0.96; *p* = 0.041); and harm you caused (OR = 1.13; *p* = 0.046). The rheumatoid arthritis-PGS was associated with two reported SLEs: fire or explosion (OR = 0.90; *p* = 0.002) and serious injury, harm, or death you caused to someone else (OR = 1.14; *p* = 0.033). Finally, the T2D-PGS was associated with 18 SLE variables, with the strongest five associations being: marital separation (OR = 1.21; *p* = 0.001); total accumulated SLEs (OR = 1.18; *p* < 0.001); harm you caused (OR = 1.18) (*p* = 0.007); sexual assault (OR = 1.09; *p* < 0.001); and major financial problems (OR = 1.09; *p* = 0.001).

## Discussion

In this cohort study of adults with depression, genetic vulnerability for several mental disorders was associated with an increased likelihood of reported exposure to SLEs. While all PGSs were associated with SLEs to varying degrees, the clearest patterns of increased likelihood were for depression and ADHD, and, to a lesser extent, schizophrenia and anxiety. By contrast, higher genetic risk for bipolar disorder was associated with *lower* odds of reported exposure to several SLEs, consistent with one study finding a lower bipolar-PGS among cases with more childhood maltreatment [45] but inconsistent with another reporting the opposite [46]. While counterintuitive, this association does not mean that genetic vulnerability to BD reduces the risk of genuine exposure to SLEs; rather, genetic vulnerability to BD might influence the way that individuals think about and respond to questions about stressors. These findings underscore the inter-dependence of diatheses and stressors in depression and also challenge the historical discourse about the subtyping of depression on the basis of exposure to SLEs (e.g., melancholic vs non-melancholic; endogenous vs reactive). The findings also bring into question the role of SLEs and other perceived traumatic experiences in diagnostic categories that rely primarily on the reporting of events (e.g., PTSD, prolonged grief disorder). While these experiences may represent a precipitant of abnormal mental states, the simple assignment of aetiologic causality to reported events may downplay the importance of other vulnerability factors that underpin variation in responses to SLEs. We discuss these findings in the context of active, passive, and evocative gene-environment correlations, and gene-environment interaction.

The *active* gene-environment correlation account views people with genetic vulnerability to depression as being more likely to generate stress by selecting themselves into environments carrying higher risk of stressors [12–15]. In support of this, some studies show that genetic risk for depression is more strongly linked to interpersonal than nonpersonal SLEs. Chance is presumed to play a more prominent causal role in nonpersonal SLEs [4, 13, 20, 47]. Here, most of the positive PGS-SLE associations were for personal SLEs (e.g., legal problems, sexual assault, physical assault). Dovetailing with other studies, the depression-PGS was mostly associated with personal SLEs [4], and notably, the ADHD-PGS was related to the highest number of personal SLEs. Conversely, the *passive* account suggests that since mental disorders are heritable, genetic vulnerability may be a (noncausal) marker of a parent’s psychopathology. Parental psychopathology could therefore be a cause of an offspring’s reported exposure to SLEs, for example, via a chaotic home environment [48] or negative parenting behaviours in childhood [49]. Third, the *evocative* account suggests that genetically-influenced traits evoke predictable patterns of response from the environment. Children may express traits (e.g., aggression, headstrong behaviour) that make parents engage in negative behaviours (e.g., emotional abuse). Relevant to our findings, we speculate that behavioural expression of genetic liability to ADHD (e.g., impulsivity) [50], depression (e.g., moodiness), or schizophrenia (e.g., behavioural disturbance) [51] could elicit negative responses from the environment, and these responses could be experienced or interpreted as SLEs.

A final interpretation is gene-environment interaction. As has been suggested for genetic risk for depression [13], genetic risk for other mental disorders (e.g., anxiety) may alter an individual’s sensitivity to the environment. For example, an individual with genetic vulnerability to anxiety may experience an event as stressful or attribute the intention of another person as threatening. Such genetic vulnerability could influence a shift from a neutral event—e.g., being touched on the arm as a greeting—to a stressful event—e.g., feeling physically assaulted. Relatedly, we have proposed a developmental pathway model that hypothesises three pathophysiological mechanisms underpinning trajectories to depression: a *neurodevelopmental* pathway leading to depression with cognitive impairment or psychotic features; a *circadian* pathway leading to atypical depression or bipolar-like presentations; and a *hyperarousal* pathway leading to the common forms of anxious-depression [25, 26, 52]. Speculating about our findings under this model, it is possible that for one person with high genetic risk for depression or anxiety, a neutral event may be experienced with hyperarousal and be recalled as a stressor, while for another person with lower genetic risk for depression or anxiety, the same event might be experienced with lower arousal and not be recalled as a stressor. Age-dependent expression of genetic risk may also be relevant. For example, a study of the *Avon Longitudinal Study of Parents and Children* reported that higher genetic risk for schizophrenia manifests in adolescence as anxiety [53]; people with genetic liability to schizophrenia might therefore experience life events as more stressful at certain developmental stages.

Our study has several limitations. First, we relied on retrospective reports of SLEs, which might be influenced by reporting/recall biases. Some SLE items were broad, subject to interpretation, and may reflect non-abusive behaviours; for instance, the childhood ‘emotional abuse’ item includes example prompts such as “yelled at in a scary way” and “not being given encouragement”, which may be particularly at risk of recall biases. Second, it is possible that participants may have conflated their experience of depression with items on the SLE checklist. Third, most of AGDS reports a severe, recurrent course of depression (only 4% reporting a single episode [27]). While our models adjusted for the number of depressive episodes, our findings might nonetheless be less relevant to individuals with less severe or recurrent depressive disorders. Fourth, we used the full distribution of each PGS; however, given that differences in SLEs could plausibly be quite different at the extremes of the PGS, in an exploratory analysis we compared the ORs for each SLE between the top and bottom 5% of each PGS. Across the six psychiatric PGS, there was a difference of less than 10% in the ORs of most SLEs, which suggests that if there are nonlinear PGS-SLE associations, they are minor. Fifth, we cannot know whether the PGS-SLE associations reflect an increase in the probability of genuine exposure to SLEs versus an increase in the attribution of events as stressors. Sixth, differences in the patterns of association between the PGS may be related to differences in the relative power of each PGS (which are influenced by GWAS sample sizes and the genetic architecture of the phenotypes). Seventh, most of AGDS is of European ancestry (> 95%) and our PGS analyses were on participants with genetic similarity to a European reference group; our adjustment for genetic PCs accounts for differences within the European group and cryptic relatedness between samples (which in one sense is a strength). However, our findings may not be generalisable to non-European groups, as reporting of SLEs (and associations with PGS) might differ across ancestry/ethnic groups. Eighth, the higher proportion of participants recruited to AGDS via public media appeal (86%) compared to prescription history invitation (14%), and the high educational attainment, may indicate a self-selection bias that restricts the generalisability of our findings. Ninth, a person’s current relationship with family could bias their reporting of childhood SLEs. The survey did not collect detailed information about relationships with family. However, using our best item (serious problems getting along with ‘other family member’ during the past 12 months), we found that although the childhood SLEs were significantly associated with this current family relationship variable (*P* < 1 × 10^−^^200^), the PGS and childhood SLE associations remained significant when adjusting for this covariate.

In conclusion, among adults with more severe or recurrent forms of depression, higher PGSs for ADHD, depression, anxiety, and schizophrenia were consistently associated with higher reporting of exposure to SLEs. Notably however, our post-hoc analysis with non-psychiatric PGSs suggest a more complex story. While the psychiatric PGS were more consistently associated with childhood SLEs, and had stronger associations with each cumulative SLE variable, the magnitude and pattern of association for the past-year/lifetime SLEs were more similar among the psychiatric and non-psychiatric PGS (particularly for the T2D-PGS). Replication and follow-up causal analyses of these post-hoc results is warranted. Altogether, our results suggest that efforts to help genetically-vulnerable individuals cope with stress could potentially improve their outcomes and help break the cycle of recurrent depression and SLEs.

## Supplementary information

Supplementary Figures

Supplementary Tables

## Data Availability

Access to AGDS data is restricted due to the ethical guidelines governing the study but may be accessible following ethical review and data transfer agreements. Please contact Nicholas Martin (nick.martin@qimrberghofer.edu.au) with queries related to accessing AGDS data.

## Code availability

R analysis scripts used to run the statistical models can be shared upon reasonable request.

## References

[1] Herrman H, Patel V, Kieling C, Berk M, Buchweitz C, Cuijpers P, et al. Time for united action on depression: a Lancet-world Psychiatric Association Commission. Lancet*.* 2022;399:957-1022 10.1016/S0140-6736(21)02141-3 35180424

[2] Monroe SM, Simons AD. Diathesis-stress theories in the context of life stress research: implications for the depressive disorders. Psychol Bull. 1991;110:406–25. 1758917 10.1037/0033-2909.110.3.406

[3] Peyrot WJ, Milaneschi Y, Abdellaoui A, Sullivan PF, Hottenga JJ, Boomsma DI, et al. Effect of polygenic risk scores on depression in childhood trauma. Br J Psychiatry. 2014;205:113–9. 24925986 10.1192/bjp.bp.113.143081 PMC4118052

[4] Colodro-Conde L, Couvy-Duchesne B, Zhu G, Coventry WL, Byrne EM, Gordon S, et al. A direct test of the diathesis–stress model for depression. Mol Psychiatry. 2018;23:1590–6. 28696435 10.1038/mp.2017.130 PMC5764823

[5] World Health Organization. World mental health report: transforming mental health for all. Geneva. 2022.

[6] Paykel ES. Basic concepts of depression. Dialogues Clin Neurosci. 2008;10:279–89. 18979941 10.31887/DCNS.2008.10.3/espaykel PMC3181879

[7] Warrier V, Kwong ASF, Luo M, Dalvie S, Croft J, Sallis HM, et al. Gene-environment correlations and causal effects of childhood maltreatment on physical and mental health: a genetically informed approach. Lancet Psychiatry. 2021;8:373–86. 33740410 10.1016/S2215-0366(20)30569-1 PMC8055541

[8] Kendler KS, Karkowski LM, Prescott CA. The assessment of dependence in the study of stressful life events: validation using a twin design. Psychol Med. 1999;29:1455–60. 10616952 10.1017/s0033291798008198

[9] Bemmels HR, Burt SA, Legrand LN, Iacono WG, McGue M. The heritability of life events: an adolescent twin and adoption study. Twin Res Hum Genet. 2008;11:257–65. 18498204 10.1375/twin.11.3.257

[10] Kendler KS, Neale M, Kessler R, Heath A, Eaves L. A twin study of recent life events and difficulties. Arch Gen Psychiatry. 1993;50:789–96. 8215803 10.1001/archpsyc.1993.01820220041005

[11] Plomin R, Lichtenstein P, Pedersen NL, McClearn GE, Nesselroade JR. Genetic influence on life events during the last half of the life span. Psychol Aging. 1990;5:25–30. 2317298 10.1037//0882-7974.5.1.25

[12] Kendler KS, Karkowski LM, Prescott CA. Causal relationship between stressful life events and the onset of major depression. Am J Psychiatry. 1999;156:837–41. 10360120 10.1176/ajp.156.6.837

[13] Kendler KS. Major depression and the environment: a psychiatric genetic perspective. Georg Thieme Verl. 1998;31:5–9. 10.1055/s-2007-979287 9524977

[14] Hammen C. Stress generation in depression: reflections on origins, research, and future directions. J Clin Psychol. 2006;62:1065–82. 16810666 10.1002/jclp.20293

[15] Liu RT, Alloy LB. Stress generation in depression: a systematic review of the empirical literature and recommendations for future study. Clin Psychol Rev. 2010;30:582–93. 20478648 10.1016/j.cpr.2010.04.010 PMC3049314

[16] Kendler KS, Baker JH. Genetic influences on measures of the environment: a systematic review. Psychol Med. 2007;37:615–26. 17176502 10.1017/S0033291706009524

[17] Arnau-Soler A, Adams MJ, Clarke T-K, MacIntyre DJ, Milburn K, Navrady L, et al. A validation of the diathesis-stress model for depression in Generation Scotland. Transl Psychiatry. 2019;9:25. 30659167 10.1038/s41398-018-0356-7 PMC6338746

[18] Musliner KL, Andersen KK, Agerbo E, Albiñana C, Vilhjalmsson BJ, Rajagopal VM, et al. Polygenic liability, stressful life events and risk for secondary-treated depression in early life: a nationwide register-based case-cohort study. Psychol Med. 2021;1–10. 10.1017/S0033291721001410 33949298

[19] Peyrot WJ, Van der Auwera S, Milaneschi Y, Dolan CV, Madden PAF, Sullivan PF, et al. Does childhood trauma moderate polygenic risk for depression? A meta-analysis of 5765 subjects from the psychiatric genomics consortium. Biol psychiatry. 2018;84:138–47. 29129318 10.1016/j.biopsych.2017.09.009 PMC5862738

[20] Mullins N, Power RA, Fisher HL, Hanscombe KB, Euesden J, Iniesta R, et al. Polygenic interactions with environmental adversity in the aetiology of major depressive disorder. Psychol Med. 2016;46:759–70. 26526099 10.1017/S0033291715002172 PMC4754832

[21] Clarke TK, Zeng Y, Navrady L, Xia C, Haley C, Campbell A, et al. Genetic and environmental determinants of stressful life events and their overlap with depression and neuroticism. Wellcome Open Res. 2018;3:11. 30756089 10.12688/wellcomeopenres.13893.1 PMC6352921

[22] Musliner KL, Seifuddin F, Judy JA, Pirooznia M, Goes FS, Zandi PP. Polygenic risk, stressful life events and depressive symptoms in older adults: a polygenic score analysis. Psychol Med. 2015;45:1709–20. 25488392 10.1017/S0033291714002839 PMC4412793

[23] Ratanatharathorn A, Koenen KC, Chibnik LB, Weisskopf MG, Rich-Edwards JW, Roberts AL. Polygenic risk for autism, attention-deficit hyperactivity disorder, schizophrenia, major depressive disorder, and neuroticism is associated with the experience of childhood abuse. Mol Psychiatry. 2021;26:1696–705. 33483690 10.1038/s41380-020-00996-w PMC8164961

[24] Peel AJ, Purves KL, Baldwin JR, Breen G, Coleman JRI, Pingault JB, et al. Genetic and early environmental predictors of adulthood self-reports of trauma. Br J Psychiatry. 2022;221:613–20 10.1192/bjp.2021.207 35105391

[25] Hickie IB, Scott EM, Cross SP, Iorfino F, Davenport TA, Guastella AJ, et al. Right care, first time: a highly personalised and measurement-based care model to manage youth mental health. Med J Aust. 2019;211:S3–s46. 31679171 10.5694/mja2.50383

[26] Hickie IB, Hermens DF, Naismith SL, Guastella AJ, Glozier N, Scott J, et al. Evaluating differential developmental trajectories to adolescent-onset mood and psychotic disorders. BMC Psychiatry. 2013;13:303. 24215120 10.1186/1471-244X-13-303 PMC4226022

[27] Byrne EM, Kirk KM, Medland SE, McGrath JJ, Colodro-Conde L, Parker R, et al. Cohort profile: the Australian genetics of depression study. BMJ Open. 2020;10:e032580. 32461290 10.1136/bmjopen-2019-032580 PMC7259831

[28] Elm EV, Altman DG, Egger M, Pocock SJ, Gøtzsche PC, Vandenbroucke JP. Strengthening the reporting of observational studies in epidemiology (STROBE) statement: guidelines for reporting observational studies. BMJ. 2007;335:806–8. 17947786 10.1136/bmj.39335.541782.AD PMC2034723

[29] Weathers FW, Blake DD, Schnurr PP, Kaloupek DG, Marx BP, Keane TM. The life events checklist for DSM-5 (LEC-5) – standard. 2013.

[30] Brugha T, Bebbington P, Tennant C, Hurry J. The list of threatening experiences: a subset of 12 life event categories with considerable long-term contextual threat. Psychol Med. 1985;15:189–94. 3991833 10.1017/s003329170002105x

[31] Abecasis GR, Altshuler D, Auton A, Brooks LD, Durbin RM, Gibbs RA, et al. A map of human genome variation from population-scale sequencing. Nature. 2010;467:1061–73. 20981092 10.1038/nature09534 PMC3042601

[32] Purcell S, Neale B, Todd-Brown K, Thomas L, Ferreira MAR, Bender D, et al. PLINK: a tool set for whole-genome association and population-based linkage analyses. Am J Hum Genet. 2007;81:559–75. 17701901 10.1086/519795 PMC1950838

[33] Chang CC, Chow CC, Tellier LC, Vattikuti S, Purcell SM, Lee JJ. Second-generation PLINK: rising to the challenge of larger and richer datasets. Gigascience. 2015;4:7. 25722852 10.1186/s13742-015-0047-8 PMC4342193

[34] National Academies of Sciences E, and Medicine. Using population descriptors in genetics and genomics research: a new framework for an evolving field. Washington, DC. 2023. 36989389

[35] Haplotype Reference Consortium. A reference panel of 64,976 haplotypes for genotype imputation. Nat Genet. 2016;48:1279–83. 27548312 10.1038/ng.3643 PMC5388176

[36] Howard DM, Adams MJ, Clarke T-K, Hafferty JD, Gibson J, Shirali M, et al. Genome-wide meta-analysis of depression identifies 102 independent variants and highlights the importance of the prefrontal brain regions. Nat Neurosci. 2019;22:343–52. 30718901 10.1038/s41593-018-0326-7 PMC6522363

[37] Stahl EA, Breen G, Forstner AJ, McQuillin A, Ripke S, Trubetskoy V, et al. Genome-wide association study identifies 30 loci associated with bipolar disorder. Nat Genet. 2019;51:793–803. 31043756 10.1038/s41588-019-0397-8 PMC6956732

[38] Pardiñas AF, Holmans P, Pocklington AJ, Escott-Price V, Ripke S, Carrera N, et al. Common schizophrenia alleles are enriched in mutation-intolerant genes and in regions under strong background selection. Nat Genet. 2018;50:381–9. 29483656 10.1038/s41588-018-0059-2 PMC5918692

[39] Purves KL, Coleman JRI, Meier SM, Rayner C, Davis KAS, Cheesman R, et al. A major role for common genetic variation in anxiety disorders. Mol Psychiatry. 2020;25:3292–303. 31748690 10.1038/s41380-019-0559-1 PMC7237282

[40] Demontis D, Walters RK, Martin J, Mattheisen M, Als TD, Agerbo E, et al. Discovery of the first genome-wide significant risk loci for attention deficit/hyperactivity disorder. Nat Genet. 2019;51:63–75. 30478444 10.1038/s41588-018-0269-7 PMC6481311

[41] Nagel M, Jansen PR, Stringer S, Watanabe K, de Leeuw CA, Bryois J, et al. Meta-analysis of genome-wide association studies for neuroticism in 449,484 individuals identifies novel genetic loci and pathways. Nat Genet. 2018;50:920–7. 29942085 10.1038/s41588-018-0151-7

[42] Lloyd-Jones LR, Zeng J, Sidorenko J, Yengo L, Moser G, Kemper KE, et al. Improved polygenic prediction by Bayesian multiple regression on summary statistics. Nat Commun. 2019;10:5086. 31704910 10.1038/s41467-019-12653-0 PMC6841727

[43] R Core Team. R: A Language and Environment for Statistical Computing. R Foundation for Statistical Computing: Vienna, Austria, 2022.

[44] Thissen D, Steinberg L, Kuang D. Quick and easy implementation of the benjamini-hochberg procedure for controlling the false positive rate in multiple comparisons. J Educ Behav Stat. 2002;27:77–83.

[45] Aas M, Bellivier F, Bettella F, Henry C, Gard S, Kahn JP, et al. Childhood maltreatment and polygenic risk in bipolar disorders. Bipolar Disord. 2020;22:174–81. 31628696 10.1111/bdi.12851

[46] Park YM, Shekhtman T, Kelsoe JR. Interaction between adverse childhood experiences and polygenic risk in patients with bipolar disorder. Transl Psychiatry. 2020;10:326. 32963226 10.1038/s41398-020-01010-1 PMC7509781

[47] Kendler KS, Karkowski-Shuman L. Stressful life events and genetic liability to major depression: genetic control of exposure to the environment? Psychol Med. 1997;27:539–47. 9153675 10.1017/s0033291797004716

[48] Agnew-Blais JC, Wertz J, Arseneault L, Belsky DW, Danese A, Pingault JB, et al. Mother’s and children’s ADHD genetic risk, household chaos and children’s ADHD symptoms: a gene-environment correlation study. J Child Psychol Psychiatry. 2022;63:1153–63. 35833717 10.1111/jcpp.13659 PMC9796059

[49] Hammen C, Shih JH, Brennan PA. Intergenerational transmission of depression: test of an interpersonal stress model in a community sample. Am Psychol Assoc. 2004;72:511–22. 10.1037/0022-006X.72.3.511 15279534

[50] Sudre G, Frederick J, Sharp W, Ishii-Takahashi A, Mangalmurti A, Choudhury S, et al. Mapping associations between polygenic risks for childhood neuropsychiatric disorders, symptoms of attention deficit hyperactivity disorder, cognition, and the brain. Mol Psychiatry. 2020;25:2482–92. 30700802 10.1038/s41380-019-0350-3 PMC6667324

[51] Jansen PR, Polderman TJC, Bolhuis K, van der Ende J, Jaddoe VWV, Verhulst FC, et al. Polygenic scores for schizophrenia and educational attainment are associated with behavioural problems in early childhood in the general population. J Child Psychol Psychiatry. 2018;59:39–47. 28627743 10.1111/jcpp.12759

[52] Crouse JJ, Carpenter JS, Song YJC, Hockey SJ, Naismith SL, Grunstein RR, et al. Circadian rhythm sleep-wake disturbances and depression in young people: implications for prevention and early intervention. Lancet Psychiatry. 2021;8:813–23. 34419186 10.1016/S2215-0366(21)00034-1

[53] Jones HJ, Stergiakouli E, Tansey KE, Hubbard L, Heron J, Cannon M, et al. Phenotypic manifestation of genetic risk for schizophrenia during adolescence in the general population. JAMA Psychiatry. 2016;73:221–8. 26818099 10.1001/jamapsychiatry.2015.3058 PMC5024747

