# Supplementary material for: Patterns of stressful life events and polygenic scores for five mental disorders and neuroticism among adults with depression
Source: Mol Psychiatry. 2024 Apr 4;29(9):2765–73. doi: 10.1038/s41380-024-02492-x (PMC11420070; doi:10.1038/s41380-024-02492-x)

**Supplementary Figure 1. PCA plots of ancestry principal components 1-3.** Participants of non-European ancestry (defined as > 4SD from the PC1 and PC2 centroid) were excluded from analyses.


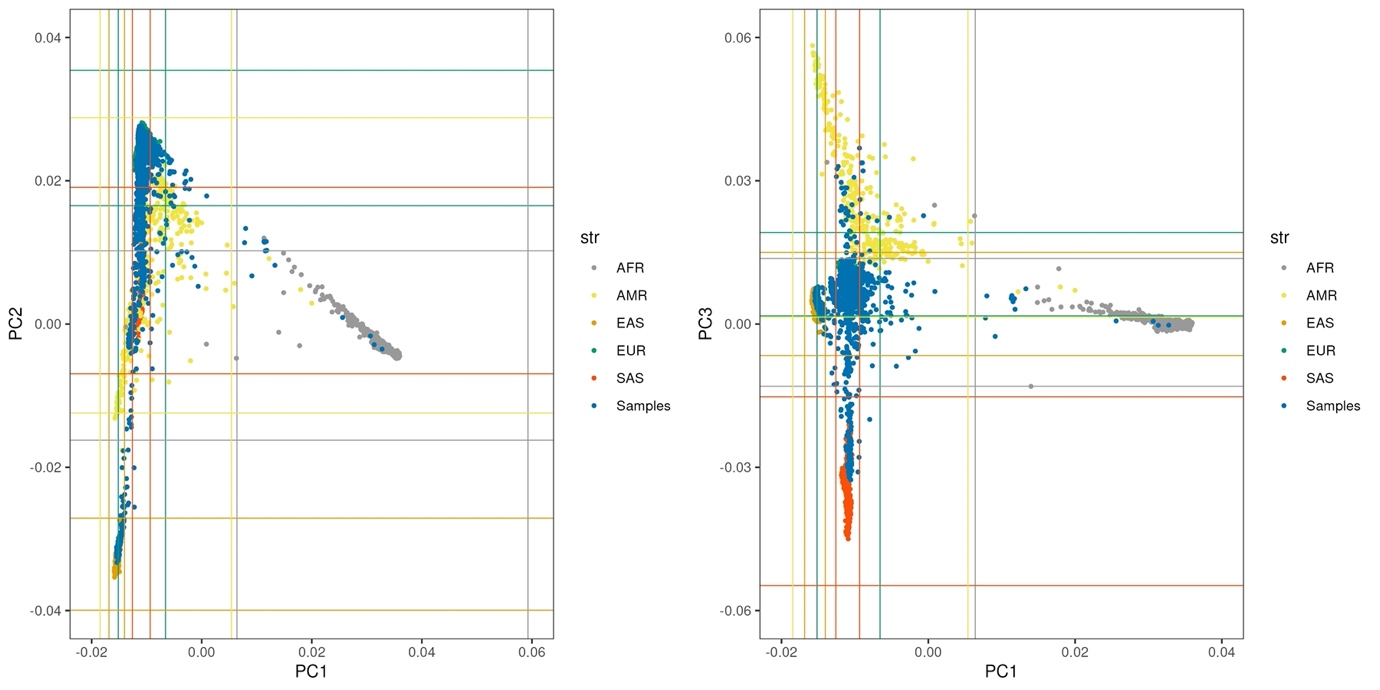


**Supplementary Figure 2. Post-hoc analysis: Childhood SLEs and non-psychiatric PGS (asthma, coronary artery disease, hip circumference, lung cancer, rheumatoid arthritis, and type 2 diabetes).** For simplicity, the coefficients are presented in groupings of PGS (not SLE). The y-axis variables are the outcome (y) variables for three regression models in which the six PGS were fitted together alongside age and sex.


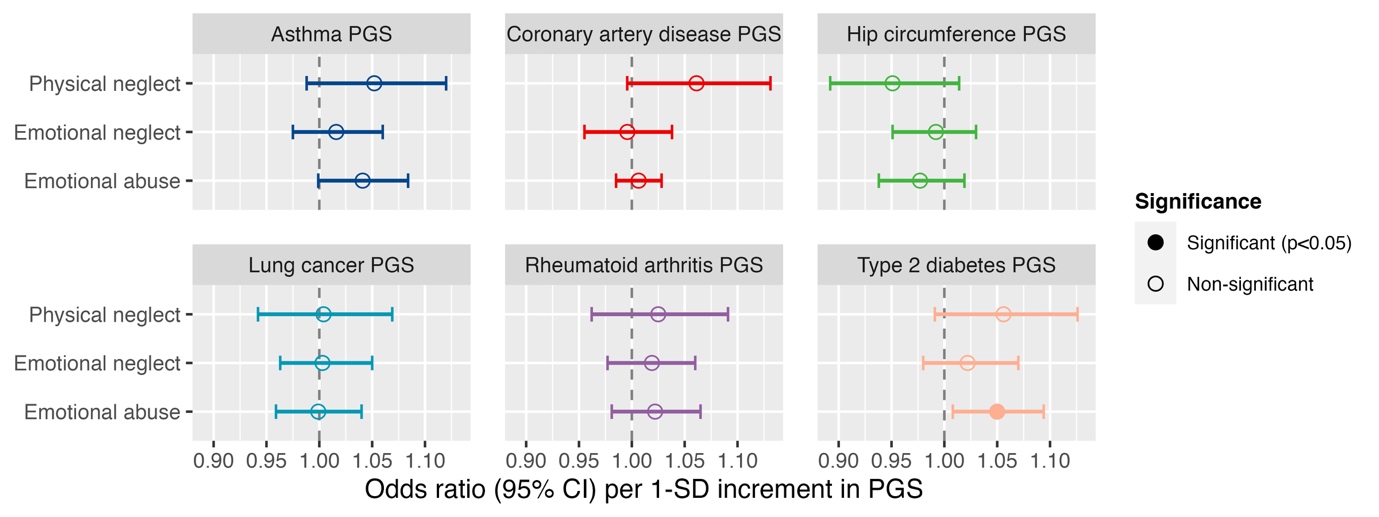


**Supplementary Figure 3. Post-hoc analysis: Lifetime SLEs and non-psychiatric PGS (asthma, coronary artery disease, hip circumference, lung cancer, rheumatoid arthritis, and type 2 diabetes).** Coefficients are presented grouped by PGS (not SLE). The y-axis variables are the outcome (y variables) for regression models in which the six PGS were fitted alongside age and sex (x variables).


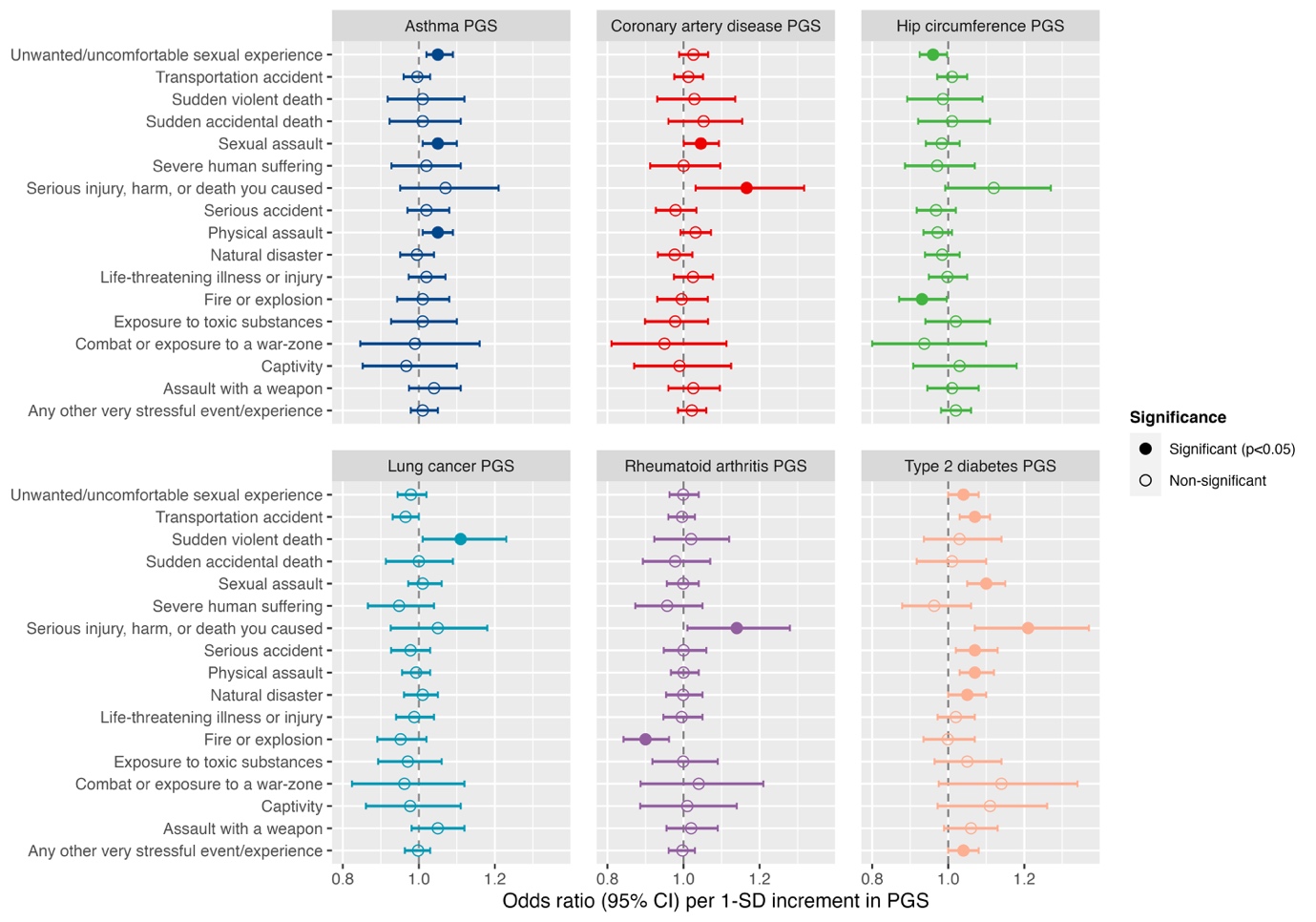


**Supplementary Figure 4. Post-hoc analysis: Past-year SLEs and non-psychiatric PGS (asthma, coronary artery disease, hip circumference, lung cancer, rheumatoid arthritis, and type 2 diabetes).** Coefficients are presented grouped by PGS (not SLE). The y-axis variables are the outcome (y variables) for regression models in which the six PGS were fitted alongside age and sex (x variables).


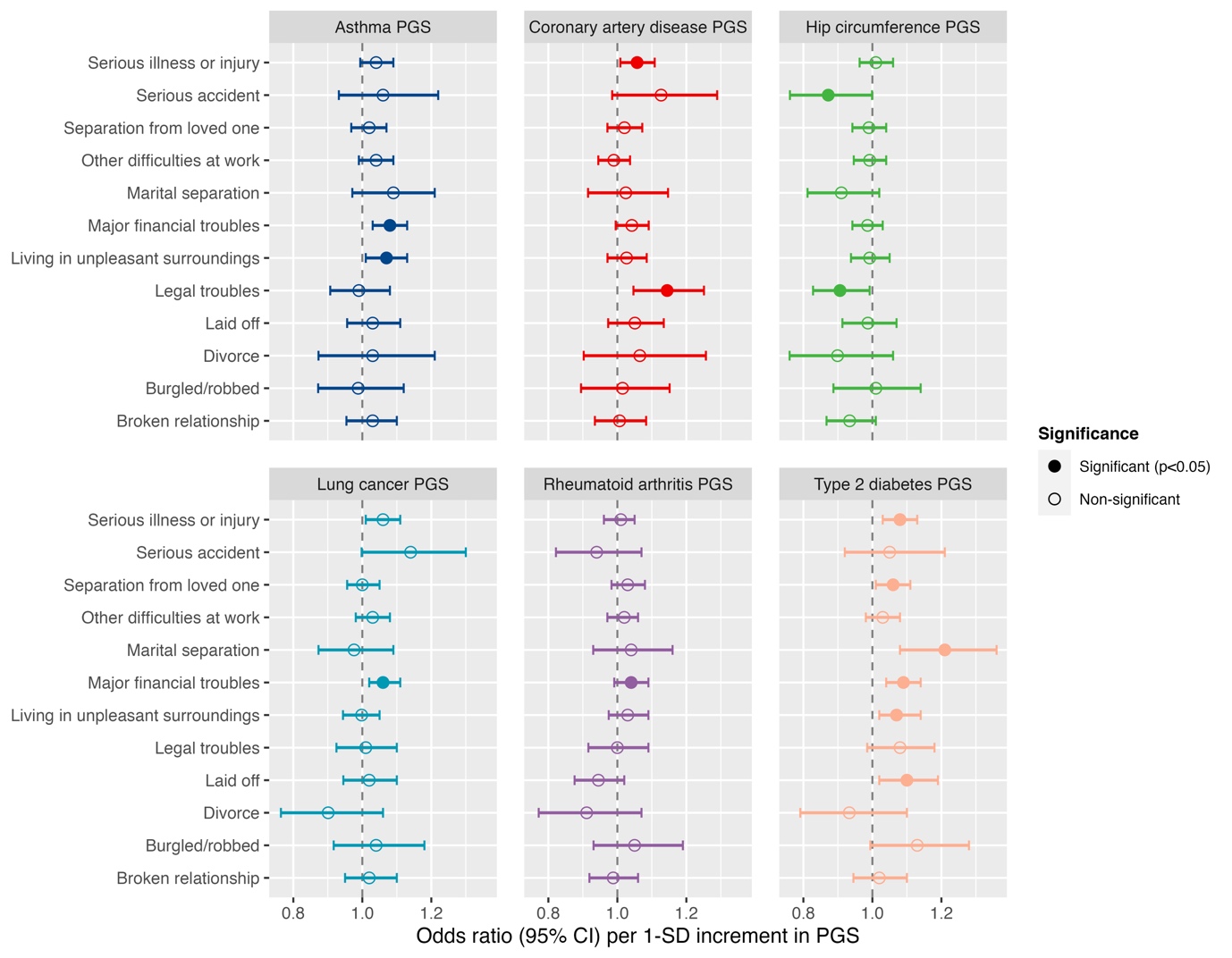


**Supplementary Figure 5. Post-hoc analysis: Cumulative SLEs and non-psychiatric PGS (asthma, coronary artery disease, hip circumference, lung cancer, rheumatoid arthritis, and type 2 diabetes).** Coefficients are presented grouped by PGS (not SLE). The y-axis variables are the outcome (y variables) for regression models in which the six PGS were fitted alongside age and sex (x variables).


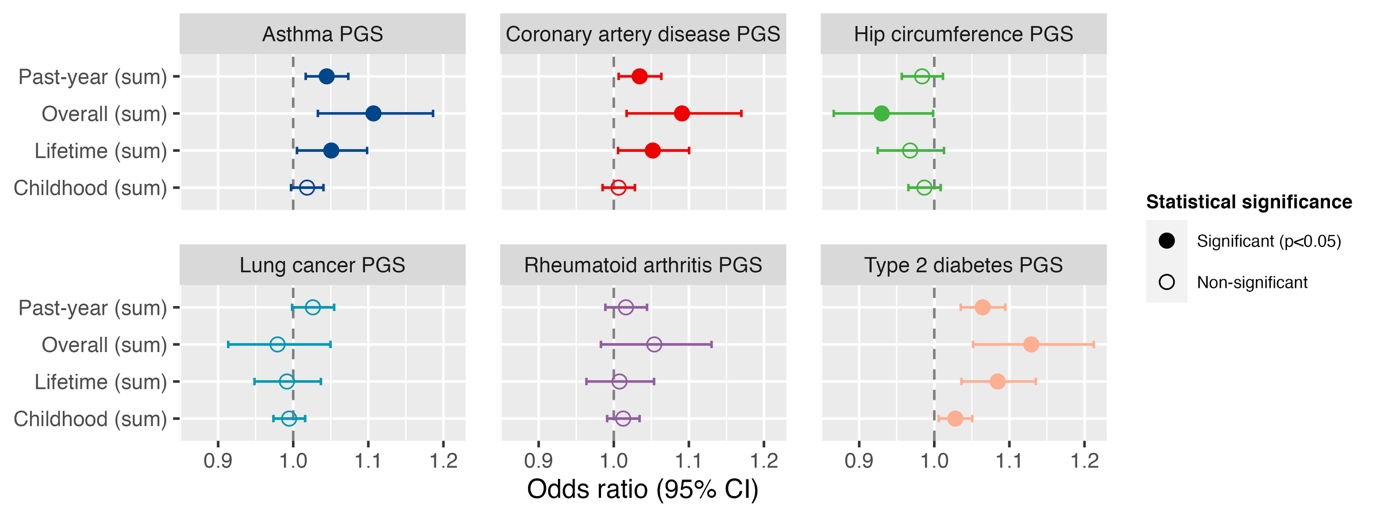

Supplement: Supplementary file 1 — Supplementary Figures [file 41380_2024_2492_MOESM1_ESM.docx]
