# Supplementary material for: Patterns of stressful life events and polygenic scores for five mental disorders and neuroticism among adults with depression
Source: Mol Psychiatry. 2024 Apr 4;29(9):2765–73. doi: 10.1038/s41380-024-02492-x (PMC11420070; doi:10.1038/s41380-024-02492-x)
Supplement: Supplementary file 2 — Supplementary Tables [file 41380_2024_2492_MOESM2_ESM.docx]

**Supplementary Table 1. Proportions of sample endorsing each SLE.**

|  | Total sample | | | Females | Males | Females vs. Males | |
| --- | --- | --- | --- | --- | --- | --- | --- |
| Childhood | N total | N endorsed | % endorsed | Endorsed (%) | | P-value | Cramer’s V |
| Emotional abuse | 10,203 | 5,375 | 53 | 54 | 50 | <0.001 | 0.04 |
| Emotional neglect | 10,076 | 4616 | 46 | 46 | 45 | 0.15 | 0.01 |
| Physical neglect | 10,623 | 1145 | 11 | 11 | 9 | 0.002 | 0.03 |
| **Past-year** |  |  |  |  |  |  |  |
| Divorce | 11,008 | 147 | 1 | 1 | 2 | 0.5 | 0.01 |
| Marital separation | 11,010 | 329 | 3 | 3 | 4 | <0.001 | 0.04 |
| Broken engagement or steady relationship | 10,991 | 819 | 7 | 8 | 7 | 0.5 | 0.01 |
| Separation from other loved one or close friend | 10,991 | 2,046 | 19 | 21 | 13 | <0.001 | 0.08 |
| Serious illness or injury | 11,003 | 2,316 | 21 | 21 | 21 | 0.7 | <0.01 |
| Serious accident (not involving personal injury) | 10,969 | 227 | 2 | 2 | 2 | 0.4 | 0.01 |
| Burgled or robbed | 11,008 | 260 | 2 | 2 | 2 | 0.9 | <0.01 |
| Laid off or sacked from job | 11,002 | 741 | 7 | 6 | 8 | <0.001 | 0.04 |
| Other serious difficulties at work | 11,004 | 2,436 | 22 | 22 | 23 | 0.4 | 0.01 |
| Major financial troubles | 11,008 | 2,505 | 23 | 23 | 21 | 0.046 | 0.02 |
| Legal troubles or involvement with police | 10,991 | 534 | 5 | 5 | 6 | 0.008 | 0.03 |
| Living in unpleasant surroundings | 10,997 | 1,560 | 14 | 14 | 14 | 0.3 | 0.01 |
| **Lifetime** |  |  |  |  |  |  |  |
| Natural disaster | 14,133 | 2,223 | 16 | 16 | 16 | 0.5 | 0.01 |
| Fire or explosion | 14,133 | 978 | 7 | 6 | 8 | <0.001 | 0.03 |
| Transportation accident | 14,133 | 4,121 | 29 | 29 | 30 | 0.093 | 0.01 |
| Serious accident | 14,133 | 1,541 | 11 | 10 | 15 | <0.001 | 0.08 |
| Exposure to toxic substances | 14,133 | 596 | 4 | 3 | 8 | <0.001 | 0.11 |
| Physical assault | 14,133 | 3,615 | 26 | 24 | 30 | <0.001 | 0.05 |
| Assault with a weapon | 14,133 | 1,005 | 7 | 6 | 10 | <0.001 | 0.06 |
| Sexual assault | 14,133 | 2,659 | 19 | 23 | 7 | <0.001 | 0.18 |
| Other unwanted or uncomfortable sexual experience | 14,133 | 4,751 | 34 | 40 | 14 | <0.001 | 0.24 |
| Combat or exposure to a warzone | 14,133 | 167 | 1 | 1 | 3 | <0.001 | 0.08 |
| Captivity | 14,133 | 247 | 2 | 2 | 1 | 0.066 | 0.02 |
| Life-threatening illness or injury | 14,133 | 1,930 | 14 | 13 | 16 | <0.001 | 0.04 |
| Severe human suffering | 14,133 | 495 | 4 | 3 | 4 | 0.001 | 0.03 |
| Sudden violent death | 14,133 | 419 | 3 | 3 | 3 | 0.2 | 0.011 |
| Sudden accidental death | 14,133 | 496 | 4 | 3 | 4 | 0.2 | 0.011 |
| Serious injury, harm, or death you caused to someone else | 14,133 | 277 | 2 | 2 | 3 | <0.001 | 0.05 |
| Any other very stressful event or experience | 14,133 | 4,498 | 32 | 33 | 28 | <0.001 | 0.05 |

**Supplementary Table 2. Unadjusted associations between polygenic scores (PGS) and stressful life events.**

|  | MDD-PGS | BD-PGS | SCZ-PGS | NEU-PGS | ANX-PGS | ADHD-PGS |
| --- | --- | --- | --- | --- | --- | --- |
| Childhood |  |  |  |  |  |  |
| Emotional abuse | .068*** | .025* | .063*** | .036*** | .063*** | .067*** |
| Emotional neglect | .080*** | .023* | .066*** | .034** | .043*** | .055*** |
| Physical neglect | .051*** | .027** | .065*** | .027** | .042*** | .049*** |
| Past-year |  |  |  |  |  |  |
| Divorce | 0.007 | 0.007 | -0.006 | 0.006 | .019* | -0.001 |
| Marital separation | 0.007 | -0.004 | 0.004 | 0.008 | 0.013 | 0.006 |
| Broken engagement or steady relationship | .024* | -0.002 | .022* | 0.018 | .024* | .019* |
| Separation from other loved one or close friend | .033** | 0.018 | 0.018 | .024* | .024* | .039*** |
| Serious illness or injury | .044*** | 0.016 | -0.004 | -0.005 | .028** | .040*** |
| Serious accident (not involving personal injury) | .028** | 0.006 | 0.01 | 0.013 | .031** | .019* |
| Burgled or robbed | 0.015 | 0.013 | 0.001 | 0.001 | -0.006 | 0.011 |
| Laid off or sacked from job | 0.011 | 0.013 | 0.016 | 0.003 | -0.002 | -0.006 |
| Other serious difficulties at work | 0.019 | 0.012 | 0.016 | 0.002 | 0.005 | 0.018 |
| Major financial troubles | .042*** | -0.013 | .020* | .037*** | .022* | .049*** |
| Legal troubles or involvement with police | 0.01 | 0.001 | .022* | .020* | 0.014 | .026** |
| Living in unpleasant surroundings | .041*** | -0.016 | 0.017 | .038*** | .019* | .023* |
| Lifetime |  |  |  |  |  |  |
| Natural disaster | 0.002 | -0.013 | -0.008 | -0.015 | 0.001 | 0.015 |
| Fire or explosion | 0.004 | -0.005 | 0.008 | < .001 | 0.007 | .023** |
| Transportation accident | 0.007 | -0.01 | -0.014 | -0.007 | .025** | .020* |
| Serious accident | 0.006 | 0.004 | 0.003 | -0.002 | 0.003 | .019* |
| Exposure to toxic substances | 0.014 | -0.007 | 0 | 0.003 | 0.006 | 0.001 |
| Physical assault | .035*** | < .001 | .018* | 0.001 | .039*** | .041*** |
| Assault with a weapon | .034*** | 0.002 | 0.007 | 0.012 | .020* | .027** |
| Sexual assault | .037*** | 0.006 | 0.015 | -0.004 | .017* | .051*** |
| Other unwanted or uncomfortable sexual experience | .036*** | 0.013 | .019* | -0.008 | .029** | .031*** |
| Combat or exposure to a warzone | 0.015 | -0.003 | 0.004 | 0.004 | -0.003 | 0.013 |
| Captivity | .024** | 0.011 | 0.013 | 0.007 | .020* | .023** |
| Life-threatening illness or injury | .034*** | 0.005 | 0.005 | 0.004 | .026** | 0.011 |
| Severe human suffering | .035*** | .027** | .036*** | -0.002 | .017* | 0.014 |
| Sudden violent death | .022** | .019* | 0.014 | 0.01 | 0.002 | .034*** |
| Sudden accidental death | < .001 | 0.011 | -0.012 | 0.01 | -0.01 | .023** |
| Serious injury, harm, or death you caused to someone else | 0.016 | 0.002 | -0.006 | 0.011 | -0.005 | 0.007 |
| Any other very stressful event or experience | .024** | .022** | .026** | 0.004 | .023** | .026** |
| Cumulative |  |  |  |  |  |  |
| Childhood (0-3) | .084*** | .028** | .084*** | .042*** | .068*** | .072*** |
| Past-year (0-12) | .063*** | 0.006 | .022* | .032** | .034*** | .060*** |
| Lifetime (0-17) | .037*** | 0.007 | 0.013 | -0.001 | .032*** | .039*** |
| Overall (0-32) | .086*** | .025* | .060*** | .038*** | .061*** | .098*** |
|  |  |  |  |  |  |  |
| Note: *p<0.05; **p<0.01; ***p<0.001 |  |  |  |  |  |  |

**Supplementary Table 3. Unadjusted associations between number of depressive episodes and polygenic scores (PGS).**

| Variable | Correlation coefficient | P-value |
| --- | --- | --- |
|  |  |  |
| Depression-PGS | 0.065 | <0.001 |
| Bipolar-PGS | 0.01 | 0.254 |
| Schizophrenia-PGS | 0.036 | <0.001 |
| Neuroticism-PGS | 0.055 | <0.001 |
|  |  |  |
| Anxiety-PGS | 0.028 | 0.002 |
| ADHD-PGS | 0.057 | <0.001 |

**Supplementary Table 4. Unadjusted associations between number of depressive episodes and SLEs.**

|  | Correlation coefficient | P-value |
| --- | --- | --- |
| Childhood |  |  |
| Emotional abuse | 0.194 | <0.001 |
| Emotional neglect | 0.19 | <0.001 |
| Physical neglect | 0.123 | <0.001 |
| Past-year |  |  |
| Divorce | 0.025 | 0.012 |
| Marital separation | 0.023 | 0.02 |
| Broken engagement or steady relationship | 0.037 | <0.001 |
| Separation from other loved one or close friend | 0.055 | <0.001 |
| Serious illness or injury | 0.054 | <0.001 |
| Serious accident (not involving personal injury) | 0.015 | 0.123 |
| Burgled or robbed | 0.016 | 0.102 |
| Laid off or sacked from job | 0.042 | <0.001 |
| Other serious difficulties at work | 0.032 | 0.001 |
| Major financial troubles | 0.116 | <0.001 |
| Legal troubles or involvement with police | 0.051 | <0.001 |
| Living in unpleasant surroundings | 0.104 | <0.001 |
| Lifetime |  |  |
| Natural disaster | 0.027 | 0.002 |
| Fire or explosion | 0.022 | 0.012 |
| Transportation accident | 0.016 | 0.068 |
| Serious accident | 0.051 | 0 |
| Exposure to toxic substances | 0.029 | 0.001 |
| Physical assault | 0.108 | <0.001 |
| Assault with a weapon | 0.07 | <0.001 |
| Sexual assault | 0.103 | <0.001 |
| Other unwanted or uncomfortable sexual experience | 0.068 | <0.001 |
| Combat or exposure to a warzone | 0.015 | 0.093 |
| Captivity | 0.041 | <0.001 |
| Life-threatening illness or injury | 0.062 | <0.001 |
| Severe human suffering | 0.06 | <0.001 |
| Sudden violent death | 0.022 | 0.012 |
| Sudden accidental death | 0.019 | 0.03 |
| Serious injury, harm, or death you caused to someone else | 0.028 | 0.001 |
| Any other very stressful event or experience | 0.055 | <0.001 |
| Cumulative |  |  |
| Childhood (0-3) | 0.225 | <0.001 |
| Past-year (0-12) | 0.12 | <0.001 |
| Lifetime (0-17) | 0.091 | <0.001 |
| Overall (0-32) | 0.227 | <0.001 |

**Supplementary Table 5. Associations with emotional abuse (childhood).**

**Main analysis**

**Sensitivity analyses**

**Supplementary Table 6. Associations with emotional neglect (childhood).**

**Main analysis**

**Sensitivity analyses**

**Supplementary Table 7. Associations with physical neglect (childhood).**

**Main analysis**

**Sensitivity analyses**

**Supplementary Table 8. Associations with accumulated childhood SLEs.**

**Main analysis**

**Sensitivity analyses**

**Supplementary Table 9. Associations with divorce (past-year).**

**Main analysis**

**Sensitivity analyses**

**Supplementary Table 10. Associations with marital separation (past-year).**

**Main analysis**

**Sensitivity analyses**

**Supplementary Table 11. Associations with broken engagement or steady relationship (past-year).**

**Main analysis**

**Sensitivity analyses**

**Supplementary Table 12. Associations with separation from other loved one or close friend (past-year).**

**Main analysis**

**Sensitivity analyses**

**Supplementary Table 13. Associations with serious illness or injury (past-year).**

**Main analysis**

**Sensitivity analyses**

**Supplementary Table 14. Associations with serious accident (not involving personal injury) (past-year).**

**Main analysis**

**Sensitivity analyses**

**Supplementary Table 15. Associations with burgled or robbed (past-year).**

**Main analysis**

**Sensitivity analyses**

**Supplementary Table 16. Associations with laid off or sacked from job (past-year).**

**Main analysis**

**Sensitivity analyses**

**Supplementary Table 17. Associations with other serious difficulties at work (past-year).**

**Main analysis**

**Sensitivity analysis**

**Supplementary Table 18. Associations with major financial troubles (past-year).**

**Main analysis**

**Sensitivity analyses**

**Supplementary Table 19. Associations with legal troubles or involvement with police (past-year).**

**Main analysis**

**Sensitivity analyses**

**Supplementary Table 20. Associations with living in unpleasant surroundings (past-year).**

**Main analysis**

**Sensitivity analysis**

**Supplementary Table 21. Accumulated past-year SLEs.**

**Main analysis**

**Sensitivity analyses**

**Supplementary Table 22. Associations with natural disaster (Lifetime).**

**Main analysis**

**Supplementary analysis**

**Supplementary Table 23. Associations with fire or explosion (Lifetime).**

**Main analysis**

**Sensitivity analyses**

**Supplementary Table 24. Associations with transportation accident (Lifetime).**

**Main analysis**

**Supplementary analyses**

**Supplementary Table 25. Associations with serious accident (Lifetime).**

**Main analysis**

**Sensitivity analyses**

**Supplementary Table 26. Associations with exposure to toxic substances (Lifetime).**

**Main analysis**

**Sensitivity analyses**

**Supplementary Table 27. Associations with physical assault (Lifetime).**

**Main analysis**

**Sensitivity analyses**

**Supplementary Table 28. Associations with assault with a weapon (Lifetime).**

**Main analysis**

**Sensitivity analyses**

**Supplementary Table 29. Associations with sexual assault (Lifetime).**

**Main analysis**

**Sensitivity analyses**

**Supplementary Table 30. Associations with other unwanted or uncomfortable sexual experience (Lifetime).**

**Main analysis**

**Sensitivity analyses**

**Supplementary Table 31. Associations with combat or exposure to a warzone (Lifetime).**

**Main analysis**

**Sensitivity analyses**

**Supplementary Table 32. Associations with captivity (Lifetime).**

**Main analysis**

**Sensitivity analyses**

**Supplementary Table 33. Associations with life-threatening illness or injury (Lifetime).**

**Main analysis**

**Sensitivity analyses**

**Supplementary Table 34. Associations with severe human suffering (Lifetime).**

**Main analysis**

**Sensitivity analyses**

**Supplementary Table 35. Associations with sudden violent death (Lifetime).**

**Main analysis**

**Sensitivity analyses**

**Supplementary Table 36. Associations with sudden accidental death (Lifetime).**

**Main analysis**

**Sensitivity analyses**

**Supplementary Table 37. Associations with serious injury, harm, or death you caused to someone else (Lifetime).**

**Main analysis**

**Sensitivity analyses**

**Supplementary Table 38. Associations with any other very stressful event or experience (Lifetime).**

**Main analysis**

**Sensitivity analyses**

**Supplementary Table 39. Associations with accumulated Lifetime SLEs.**

**Main analysis**

**Sensitivity analyses**

**Supplementary Table 40. Associations with total accumulated SLEs.**

**Main analysis**

**Sensitivity analyses**
